# 1-Bromo­acetyl-2,6-bis­(4-methoxy­phen­yl)-3,5-dimethyl­piperidin-4-one

**DOI:** 10.1107/S1600536808030213

**Published:** 2008-09-27

**Authors:** R. Ramachandran, G. Aridoss, D. Velmurugan, S. Kabilan, Y. T. Jeong

**Affiliations:** a Department of Chemistry, Annamalai University, Annamalai Nagar 608 002, India; b Division of Image and Information Engineering, Pukyong National University, Busan 608-739, Republic of Korea; c Centre of Advanced Study in Crystallography and Biophysics, University of Madras, Guindy Campus, Chennai 600 025, India

## Abstract

In the title compound, C_23_H_26_BrNO_4_, the piperidinone ring adopts a boat conformation. The dihedral angle between the two benzene rings is 70.9 (1)°. The two meth­oxy groups are close to coplanar with the attached benzene rings [C—C—O—C torsion angles of 6.3 (5) and 16.4 (4)°]. A weak C—H⋯Br intra­molecular inter­action is observed. In the crystal structure, mol­ecules are linked into a chain along [101] by inter­molecular C—H⋯O hydrogen bonds. A short inter­molecular Br⋯O contact [3.063 (2) Å] is observed.

## Related literature

For background on the piperidine ring system, see: O’Hagan (2000 ▶); Pinder (1992 ▶). For information on the aryl­piperidine scaffold, see: Horton *et al.* (2003 ▶). For piperidone derivatives, see: Baluja *et al.* (1964 ▶); Mutus *et al.* (1989 ▶). For the biological activities of compounds possessing an amide bond linkage, see: Priya *et al.* (2007 ▶); Bylov *et al.* (1999 ▶); Dollery (1999 ▶). For the activivities of chloro­acetyl and heterocyclicacetyl derivatives of variously functionalized 2,6-diaryl­piperidin-4-ones, see: Aridoss *et al.* (2007*a* ▶,*b* ▶; 2008*a* ▶). For a related structure, see: Aridoss *et al.* (2008*b* ▶). For ring conformational analysis, see: Cremer & Pople (1975 ▶); Nardelli (1983 ▶).
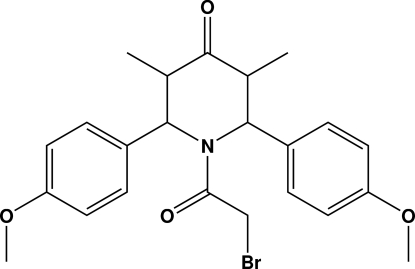


## Experimental

### 

#### Crystal data

C_23_H_26_BrNO_4_
*M* *_r_* = 460.36
Monoclinic, 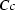

*a* = 12.9487 (9) Å
*b* = 25.2882 (18) Å
*c* = 8.9701 (6) Å
β = 132.930 (1)°
*V* = 2150.6 (3) Å^3^
*Z* = 4
Mo *K*α radiation
μ = 1.94 mm^−1^
*T* = 293 (2) K
0.30 × 0.20 × 0.16 mm

#### Data collection

Bruker Kappa APEXII diffractometer
Absorption correction: multi-scan (*SADABS*; Bruker, 1999 ▶) *T*_min_ = 0.594, *T*_max_ = 0.747
13660 measured reflections
5139 independent reflections
3748 reflections with *I* > 2σ(*I*)
*R*_int_ = 0.023

#### Refinement

*R*[*F* ^2^ > 2σ(*F* ^2^)] = 0.033
*wR*(*F* ^2^) = 0.098
*S* = 1.02
5139 reflections
266 parameters
2 restraints
H-atom parameters constrained
Δρ_max_ = 0.42 e Å^−3^
Δρ_min_ = −0.22 e Å^−3^
Absolute structure: Flack (1983 ▶), 1651 Friedel pairs
Flack parameter: 0.004 (7)

### 

Data collection: *APEX2* (Bruker, 2004 ▶); cell refinement: *SAINT* (Bruker, 2004 ▶); data reduction: *SAINT*; program(s) used to solve structure: *SIR92* (Altomare *et al.*, 1993 ▶); program(s) used to refine structure: *SHELXL97* (Sheldrick, 2008 ▶); molecular graphics: *PLATON* (Spek, 2003 ▶); software used to prepare material for publication: *SHELXL97*.

## Supplementary Material

Crystal structure: contains datablocks I, global. DOI: 10.1107/S1600536808030213/ci2672sup1.cif

Structure factors: contains datablocks I. DOI: 10.1107/S1600536808030213/ci2672Isup2.hkl

Additional supplementary materials:  crystallographic information; 3D view; checkCIF report

## Figures and Tables

**Table 1:** Hydrogen-bond geometry (Å, °)

*D*—H⋯*A*	*D*—H	H⋯*A*	*D*⋯*A*	*D*—H⋯*A*
C1—H1⋯Br1	0.98	2.82	3.523 (3)	129
C20—H20*C*⋯O1^i^	0.96	2.60	3.357 (7)	136

Symmetry code: (i) 
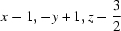
.

**Fig. 1.**
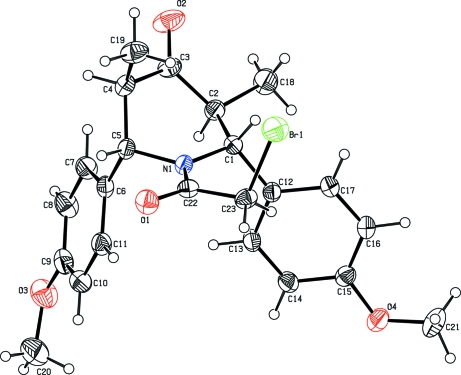
The molecular structure of title compound, showing 30% probability displacement ellipsoids.

**Fig. 2.**
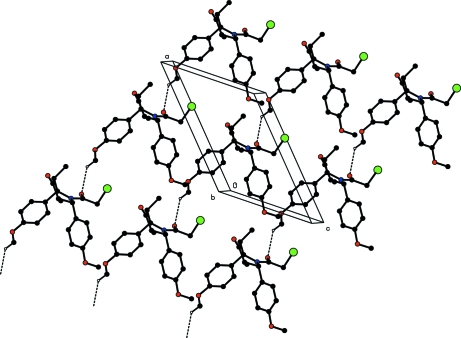
The crystal packing of the title compound. H atoms not involved in hydrogen bonding (dashed lines) have been omitted for clarity.

## supplementary crystallographic information

### Comment

The piperidine ring system is ubiquitous structural component of naturally occurring alkaloid and pharmaceuticals (O'Hagan, 2000; Pinder, 1992). Its biological properties are highly dependent on the type and location of substituents on the heterocyclic ring. The arylpiperidine scaffold is a key element involved in binding to a variety of receptors and therefore can be described as a privileged structure (Horton *et al.*, 2003). Similarly, piperidone derivatives have also received wide interest among chemists and biologists due to their envisaged mode of interaction with cellular thiols, with modest or no affinity for the hydroxy and amine groups found in nucleic acids (Baluja *et al.*, 1964; Mutus *et al.*, 1989). Generally, compounds possessing an amide bond linkage have a wide range of biological activities such as antimicrobial (Priya *et al.*, 2007), anti-inflammatory (Bylov *et al.*, 1999), antiviral, antimalarial and general anesthetics (Dollery, 1999). Recently, we have explored the antimicrobial, analgesic and antipyretic activities associated with chloroacetyl and heterocyclicacetyl derivatives of variously functionalized 2,6-diarylpiperidin-4-ones besides the change in piperidone ring conformation (Aridoss *et al.*, 2007*a*,*b*, 2008*a*). Thus, it has spurred our interest to synthesize diversely substituted 2,6-diarylpiperidin-4-ones and their derivatives. In order to establish the change in molecular conformation of piperidone ring upon bromoacetylation, the present investigation was made and confirmed by X-ray diffraction study.

The bond lengths and angles in the title molecule (Fig.1) are comparable to those observed in a related structure (Aridoss *et al.*, 2008*b*). The sum of the angles at N1 (359.0 (6)°) is in accordance with *sp*^2^ hybridization. The decrease in the N1—C22 bond length (1.368 (3) Å) when compared to C1—N1 (1.481 (3) Å) and C5—N1 (1.481 (3) Å) lengths indicates the effective conjugation between lone pair of nitrogen with carbonyl group. The N-COCH_2_ group is coplanar as confirmed by the torsion angles C1—N1—C22—C23 of -4.0 (4)° and C5—N1—C22—O1 of -172.4 (2)°. The dihedral angle between the two benzene rings is 70.9 (1)°. The C10—C9—O3—C20 (6.3 (5)°) and C16—C15—O4—C21 (16.4 (4)°) torsion angles indicate that the methoxy groups almost lie in the plane of the phenyl rings C6—C11 and C12—C17, respectively, to which they are attached.

The piperidinone ring adopts a boat conformation with the puckering parameters (Cremer & Pople, 1975) and the smallest displacement asymmetry parameters (Nardelli, 1983) being q_2_ = 0.673 (4) Å, q_3_ = -0.051 (4) Å, Q_T_ = 0.675 (4)Å, θ = 94.3 (3)° and ΔC_s_(C2) = 10.0 (3)°. A weak C—H···Br intramolecular interaction is observed in the molecular structure. In the crystal packing, the molecules are linked into a chain along [101] by intermolecular C—H···O hydrogen bonds (Fig. 2). A short intermolecular Br1···O4 (1+x, y, 1+z) contact of 3.063 (2) Å has been observed.

### Experimental

The title compound was obtained by adopting our earlier method (Aridoss *et al.*, 2007*a*). To a well stirred solution of 3,5-dimethyl-2,6-bis(*p*-methoxyphenyl)piperidin-4-one (1 equiv.) and triethylamine (1 equiv.) in freshly distilled benzene, bromoacetyl chloride (1 equiv.) in benzene was added in drop wise through the addition funnel for about half an hour. Stirring was continued until the completion of reaction. Later, it was poured into water and extracted with DCM. The combined DCM extracts was then washed well with 3% sodium bicarbonate solution and dried over anhydrous sodium sulfate. This upon evaporation and subsequent recrystallization in distilled ethanol furnished the diffraction-quality crystals of the title compound.

### Refinement

H atoms were positioned geometrically and refined using a riding model, with aromatic C-H = 0.93 Å, methine C-H = 0.98 Å, methylene C-H = 0.97 Å and methyl C-H = 0.96 Å. The U_iso_ values were set at 1.5*U*_eq_(C) for methyl H atoms and 1.2*U*_eq_(C) for the other H atoms.

### Crystal data

**Table:**

C_23_H_26_BrNO_4_	*F*(000) = 952
*M**_r_* = 460.36	*D*_x_ = 1.422 Mg m^−^^3^
Monoclinic, *C**c*	Mo *K*α radiation, λ = 0.71073 Å
Hall symbol: C -2yc	Cell parameters from 5872 reflections
*a* = 12.9487 (9) Å	θ = 1.6–31.2°
*b* = 25.2882 (18) Å	µ = 1.94 mm^−^^1^
*c* = 8.9701 (6) Å	*T* = 293 K
β = 132.930 (1)°	Prism, colourless
*V* = 2150.6 (3) Å^3^	0.30 × 0.20 × 0.16 mm
*Z* = 4	

### Data collection

**Table:**

Bruker Kappa APEXII CCD diffractometer	5139 independent reflections
Radiation source: fine-focus sealed tube	3748 reflections with *I* > 2σ(*I*)
graphite	*R*_int_ = 0.023
ω and φ scans	θ_max_ = 31.2°, θ_min_ = 1.6°
Absorption correction: multi-scan (SADABS; Bruker, 1999)	*h* = −11→18
*T*_min_ = 0.594, *T*_max_ = 0.747	*k* = −36→34
13660 measured reflections	*l* = −13→6

### Refinement

**Table:**

Refinement on *F*^2^	Secondary atom site location: difference Fourier map
Least-squares matrix: full	Hydrogen site location: inferred from neighbouring sites
*R*[*F*^2^ > 2σ(*F*^2^)] = 0.033	H-atom parameters constrained
*wR*(*F*^2^) = 0.098	*w* = 1/[σ^2^(*F*_o_^2^) + (0.0517*P*)^2^] where *P* = (*F*_o_^2^ + 2*F*_c_^2^)/3
*S* = 1.02	(Δ/σ)_max_ = 0.001
5139 reflections	Δρ_max_ = 0.42 e Å^−^^3^
266 parameters	Δρ_min_ = −0.22 e Å^−^^3^
2 restraints	Absolute structure: Flack (1983), 1651 Friedel pairs
Primary atom site location: structure-invariant direct methods	Flack parameter: 0.004 (7)

### Special details

**Table:**

Geometry. All e.s.d.'s (except the e.s.d. in the dihedral angle between two l.s. planes) are estimated using the full covariance matrix. The cell e.s.d.'s are taken into account individually in the estimation of e.s.d.'s in distances, angles and torsion angles; correlations between e.s.d.'s in cell parameters are only used when they are defined by crystal symmetry. An approximate (isotropic) treatment of cell e.s.d.'s is used for estimating e.s.d.'s involving l.s. planes.
Refinement. Refinement of *F*^2^ against ALL reflections. The weighted *R*-factor *wR* and goodness of fit *S* are based on *F*^2^, conventional *R*-factors *R* are based on *F*, with *F* set to zero for negative *F*^2^. The threshold expression of *F*^2^ > σ(*F*^2^) is used only for calculating *R*-factors(gt) *etc*. and is not relevant to the choice of reflections for refinement. *R*-factors based on *F*^2^ are statistically about twice as large as those based on *F*, and *R*- factors based on ALL data will be even larger.

### Fractional atomic coordinates and isotropic or equivalent isotropic displacement parameters (Å^2^)

**Table:**

	*x*	*y*	*z*	*U*_iso_*/*U*_eq_	
Br1	0.66353 (4)	0.384464 (14)	1.04369 (5)	0.06848 (12)	
O1	0.5273 (2)	0.48401 (7)	0.6690 (3)	0.0532 (5)	
O2	0.6012 (3)	0.29025 (9)	0.3780 (5)	0.0771 (8)	
O3	−0.0290 (3)	0.45775 (10)	−0.4164 (4)	0.0731 (7)	
O4	−0.0766 (2)	0.31531 (9)	0.3554 (3)	0.0559 (5)	
N1	0.4607 (2)	0.40235 (8)	0.5255 (3)	0.0345 (4)	
C1	0.4291 (3)	0.34626 (9)	0.5282 (4)	0.0364 (5)	
H1	0.5069	0.3326	0.6654	0.044*	
C2	0.4229 (3)	0.31286 (10)	0.3780 (4)	0.0416 (5)	
H2	0.3356	0.3223	0.2401	0.050*	
C3	0.5436 (3)	0.32436 (11)	0.3917 (4)	0.0461 (6)	
C4	0.5906 (4)	0.38105 (11)	0.4261 (6)	0.0446 (7)	
H4	0.6004	0.3901	0.3300	0.054*	
C5	0.4839 (3)	0.41869 (9)	0.3917 (4)	0.0381 (5)	
H5	0.5306	0.4532	0.4424	0.046*	
C6	0.3462 (3)	0.42782 (9)	0.1749 (4)	0.0393 (5)	
C7	0.3089 (3)	0.40271 (11)	0.0059 (4)	0.0486 (6)	
H7	0.3699	0.3780	0.0241	0.058*	
C8	0.1835 (4)	0.41381 (12)	−0.1874 (5)	0.0561 (7)	
H8	0.1600	0.3961	−0.2979	0.067*	
C9	0.0921 (3)	0.45079 (11)	−0.2196 (4)	0.0513 (7)	
C10	0.1278 (3)	0.47738 (12)	−0.0556 (5)	0.0516 (7)	
H10	0.0682	0.5031	−0.0752	0.062*	
C11	0.2541 (3)	0.46518 (10)	0.1394 (4)	0.0455 (6)	
H11	0.2772	0.4828	0.2497	0.055*	
C12	0.2944 (3)	0.33861 (9)	0.4838 (4)	0.0364 (5)	
C13	0.1691 (3)	0.36284 (11)	0.3213 (4)	0.0432 (6)	
H13	0.1668	0.3853	0.2371	0.052*	
C14	0.0474 (3)	0.35416 (10)	0.2822 (4)	0.0447 (6)	
H14	−0.0360	0.3709	0.1725	0.054*	
C15	0.0489 (3)	0.32085 (10)	0.4052 (4)	0.0408 (5)	
C16	0.1720 (4)	0.29517 (10)	0.5642 (5)	0.0492 (6)	
H16	0.1732	0.2717	0.6453	0.059*	
C17	0.2921 (3)	0.30430 (11)	0.6021 (5)	0.0488 (6)	
H17	0.3747	0.2870	0.7105	0.059*	
C18	0.4147 (5)	0.25375 (12)	0.4064 (7)	0.0680 (9)	
H18A	0.4945	0.2439	0.5445	0.102*	
H18B	0.4151	0.2337	0.3159	0.102*	
H18C	0.3294	0.2466	0.3765	0.102*	
C19	0.7355 (4)	0.38566 (13)	0.6430 (7)	0.0598 (10)	
H19A	0.7995	0.3610	0.6607	0.090*	
H19B	0.7269	0.3779	0.7389	0.090*	
H19C	0.7708	0.4210	0.6653	0.090*	
C20	−0.1223 (5)	0.49859 (18)	−0.4644 (7)	0.0873 (12)	
H20A	−0.0771	0.5322	−0.4334	0.131*	
H20B	−0.1470	0.4941	−0.3857	0.131*	
H20C	−0.2060	0.4971	−0.6068	0.131*	
C21	−0.0739 (4)	0.29285 (15)	0.5013 (6)	0.0656 (9)	
H21A	−0.0047	0.3107	0.6291	0.098*	
H21B	−0.0501	0.2560	0.5174	0.098*	
H21C	−0.1650	0.2965	0.4571	0.098*	
C22	0.4945 (3)	0.43860 (10)	0.6661 (4)	0.0384 (5)	
C23	0.4904 (3)	0.42170 (11)	0.8232 (4)	0.0434 (6)	
H23A	0.4100	0.3987	0.7608	0.052*	
H23B	0.4802	0.4525	0.8766	0.052*	

### Atomic displacement parameters (Å^2^)

**Table:**

	*U*^11^	*U*^22^	*U*^33^	*U*^12^	*U*^13^	*U*^23^
Br1	0.05974 (18)	0.0974 (3)	0.04260 (14)	0.01855 (19)	0.03263 (13)	0.01594 (17)
O1	0.0652 (13)	0.0408 (10)	0.0590 (12)	−0.0060 (9)	0.0444 (11)	−0.0084 (9)
O2	0.0764 (17)	0.0581 (13)	0.123 (2)	−0.0063 (11)	0.0782 (18)	−0.0271 (13)
O3	0.0637 (15)	0.0801 (15)	0.0478 (12)	0.0042 (12)	0.0271 (12)	0.0110 (11)
O4	0.0489 (12)	0.0669 (13)	0.0613 (12)	−0.0003 (9)	0.0412 (11)	0.0063 (10)
N1	0.0395 (11)	0.0334 (9)	0.0376 (10)	−0.0026 (8)	0.0290 (9)	−0.0018 (8)
C1	0.0388 (12)	0.0350 (12)	0.0381 (12)	0.0030 (10)	0.0272 (11)	0.0031 (9)
C2	0.0473 (14)	0.0346 (12)	0.0520 (14)	−0.0020 (10)	0.0374 (13)	−0.0035 (11)
C3	0.0457 (15)	0.0475 (15)	0.0512 (14)	0.0007 (12)	0.0354 (13)	−0.0075 (12)
C4	0.0461 (19)	0.0498 (16)	0.0493 (19)	−0.0023 (11)	0.0370 (17)	−0.0005 (12)
C5	0.0482 (15)	0.0341 (12)	0.0449 (13)	−0.0028 (10)	0.0369 (13)	−0.0007 (10)
C6	0.0513 (15)	0.0337 (12)	0.0431 (12)	−0.0033 (10)	0.0362 (12)	0.0015 (9)
C7	0.0624 (18)	0.0442 (13)	0.0469 (15)	0.0056 (12)	0.0402 (15)	0.0020 (11)
C8	0.073 (2)	0.0501 (16)	0.0428 (14)	0.0030 (15)	0.0384 (16)	0.0000 (12)
C9	0.0532 (17)	0.0517 (16)	0.0436 (14)	−0.0031 (13)	0.0308 (14)	0.0089 (12)
C10	0.0560 (18)	0.0474 (15)	0.0543 (16)	0.0074 (13)	0.0387 (15)	0.0074 (12)
C11	0.0581 (17)	0.0417 (14)	0.0470 (14)	0.0004 (12)	0.0399 (14)	0.0006 (11)
C12	0.0423 (14)	0.0329 (11)	0.0405 (12)	−0.0008 (10)	0.0307 (11)	0.0005 (9)
C13	0.0485 (15)	0.0404 (13)	0.0439 (13)	−0.0005 (11)	0.0327 (13)	0.0079 (11)
C14	0.0392 (14)	0.0450 (16)	0.0425 (13)	0.0041 (11)	0.0250 (12)	0.0089 (11)
C15	0.0422 (14)	0.0377 (13)	0.0486 (14)	−0.0060 (11)	0.0333 (13)	−0.0068 (11)
C16	0.0549 (16)	0.0480 (14)	0.0563 (17)	0.0030 (14)	0.0424 (15)	0.0127 (13)
C17	0.0492 (16)	0.0482 (15)	0.0551 (16)	0.0106 (12)	0.0379 (15)	0.0188 (13)
C18	0.099 (3)	0.0367 (15)	0.105 (3)	−0.0042 (15)	0.084 (3)	−0.0081 (16)
C19	0.043 (2)	0.060 (2)	0.068 (3)	−0.0046 (13)	0.035 (2)	−0.0127 (15)
C20	0.076 (3)	0.091 (3)	0.078 (3)	0.021 (2)	0.046 (2)	0.030 (2)
C21	0.066 (2)	0.087 (2)	0.065 (2)	−0.0187 (17)	0.0529 (19)	−0.0098 (17)
C22	0.0332 (12)	0.0415 (13)	0.0373 (12)	0.0018 (10)	0.0227 (11)	−0.0022 (10)
C23	0.0412 (14)	0.0541 (16)	0.0376 (12)	0.0032 (11)	0.0278 (12)	−0.0022 (11)

### Geometric parameters (Å, °)

**Table:**

Br1—C23	1.945 (3)	C10—C11	1.390 (4)
O1—C22	1.219 (3)	C10—H10	0.93
O2—C3	1.201 (3)	C11—H11	0.93
O3—C9	1.354 (4)	C12—C13	1.382 (4)
O3—C20	1.415 (5)	C12—C17	1.387 (4)
O4—C15	1.367 (3)	C13—C14	1.379 (4)
O4—C21	1.406 (4)	C13—H13	0.93
N1—C22	1.368 (3)	C14—C15	1.377 (4)
N1—C5	1.481 (3)	C14—H14	0.93
N1—C1	1.481 (3)	C15—C16	1.378 (4)
C1—C12	1.513 (3)	C16—C17	1.366 (5)
C1—C2	1.546 (3)	C16—H16	0.93
C1—H1	0.9800	C17—H17	0.93
C2—C3	1.509 (4)	C18—H18A	0.96
C2—C18	1.532 (4)	C18—H18B	0.96
C2—H2	0.98	C18—H18C	0.96
C3—C4	1.505 (4)	C19—H19A	0.96
C4—C5	1.526 (4)	C19—H19B	0.96
C4—C19	1.532 (4)	C19—H19C	0.96
C4—H4	0.98	C20—H20A	0.96
C5—C6	1.514 (4)	C20—H20B	0.96
C5—H5	0.98	C20—H20C	0.96
C6—C11	1.378 (4)	C21—H21A	0.96
C6—C7	1.392 (4)	C21—H21B	0.96
C7—C8	1.374 (4)	C21—H21C	0.96
C7—H7	0.93	C22—C23	1.507 (4)
C8—C9	1.375 (5)	C23—H23A	0.97
C8—H8	0.93	C23—H23B	0.97
C9—C10	1.380 (4)		
			
C9—O3—C20	118.8 (3)	C13—C12—C1	122.4 (2)
C15—O4—C21	117.8 (2)	C17—C12—C1	120.0 (2)
C22—N1—C5	116.5 (2)	C14—C13—C12	121.0 (2)
C22—N1—C1	123.0 (2)	C14—C13—H13	119.5
C5—N1—C1	119.5 (2)	C12—C13—H13	119.5
N1—C1—C12	113.59 (19)	C15—C14—C13	120.2 (2)
N1—C1—C2	111.07 (19)	C15—C14—H14	119.9
C12—C1—C2	109.6 (2)	C13—C14—H14	119.9
N1—C1—H1	107.5	O4—C15—C14	115.8 (2)
C12—C1—H1	107.5	O4—C15—C16	124.7 (2)
C2—C1—H1	107.5	C14—C15—C16	119.5 (3)
C3—C2—C18	111.1 (2)	C17—C16—C15	119.7 (3)
C3—C2—C1	112.8 (2)	C17—C16—H16	120.1
C18—C2—C1	110.9 (2)	C15—C16—H16	120.1
C3—C2—H2	107.3	C16—C17—C12	122.0 (3)
C18—C2—H2	107.3	C16—C17—H17	119.0
C1—C2—H2	107.3	C12—C17—H17	119.0
O2—C3—C4	120.8 (3)	C2—C18—H18A	109.5
O2—C3—C2	122.3 (3)	C2—C18—H18B	109.5
C4—C3—C2	116.9 (2)	H18A—C18—H18B	109.5
C3—C4—C5	111.6 (3)	C2—C18—H18C	109.5
C3—C4—C19	107.9 (3)	H18A—C18—H18C	109.5
C5—C4—C19	111.5 (2)	H18B—C18—H18C	109.5
C3—C4—H4	108.6	C4—C19—H19A	109.5
C5—C4—H4	108.6	C4—C19—H19B	109.5
C19—C4—H4	108.6	H19A—C19—H19B	109.5
N1—C5—C6	111.9 (2)	C4—C19—H19C	109.5
N1—C5—C4	108.5 (2)	H19A—C19—H19C	109.5
C6—C5—C4	117.8 (2)	H19B—C19—H19C	109.5
N1—C5—H5	105.9	O3—C20—H20A	109.5
C6—C5—H5	105.9	O3—C20—H20B	109.5
C4—C5—H5	105.9	H20A—C20—H20B	109.5
C11—C6—C7	117.2 (3)	O3—C20—H20C	109.5
C11—C6—C5	118.5 (2)	H20A—C20—H20C	109.5
C7—C6—C5	124.2 (2)	H20B—C20—H20C	109.5
C8—C7—C6	121.1 (3)	O4—C21—H21A	109.5
C8—C7—H7	119.4	O4—C21—H21B	109.5
C6—C7—H7	119.4	H21A—C21—H21B	109.5
C7—C8—C9	120.8 (3)	O4—C21—H21C	109.5
C7—C8—H8	119.6	H21A—C21—H21C	109.5
C9—C8—H8	119.6	H21B—C21—H21C	109.5
O3—C9—C8	115.4 (3)	O1—C22—N1	122.6 (2)
O3—C9—C10	125.2 (3)	O1—C22—C23	118.8 (2)
C8—C9—C10	119.4 (3)	N1—C22—C23	118.6 (2)
C9—C10—C11	119.1 (3)	C22—C23—Br1	109.78 (17)
C9—C10—H10	120.5	C22—C23—H23A	109.7
C11—C10—H10	120.5	Br1—C23—H23A	109.7
C6—C11—C10	122.3 (3)	C22—C23—H23B	109.7
C6—C11—H11	118.9	Br1—C23—H23B	109.7
C10—C11—H11	118.9	H23A—C23—H23B	108.2
C13—C12—C17	117.5 (2)		
			
C22—N1—C1—C12	66.9 (3)	C20—O3—C9—C8	−174.0 (3)
C5—N1—C1—C12	−125.1 (2)	C20—O3—C9—C10	6.3 (5)
C22—N1—C1—C2	−169.1 (2)	C7—C8—C9—O3	−179.1 (3)
C5—N1—C1—C2	−1.1 (3)	C7—C8—C9—C10	0.6 (4)
N1—C1—C2—C3	45.3 (3)	O3—C9—C10—C11	178.2 (3)
C12—C1—C2—C3	171.6 (2)	C8—C9—C10—C11	−1.6 (4)
N1—C1—C2—C18	170.6 (2)	C7—C6—C11—C10	0.8 (4)
C12—C1—C2—C18	−63.1 (3)	C5—C6—C11—C10	177.7 (3)
C18—C2—C3—O2	16.2 (4)	C9—C10—C11—C6	0.9 (4)
C1—C2—C3—O2	141.4 (3)	N1—C1—C12—C13	48.1 (3)
C18—C2—C3—C4	−163.2 (3)	C2—C1—C12—C13	−76.8 (3)
C1—C2—C3—C4	−38.0 (3)	N1—C1—C12—C17	−135.1 (2)
O2—C3—C4—C5	167.3 (3)	C2—C1—C12—C17	100.1 (3)
C2—C3—C4—C5	−13.3 (4)	C17—C12—C13—C14	1.7 (4)
O2—C3—C4—C19	−69.9 (4)	C1—C12—C13—C14	178.6 (2)
C2—C3—C4—C19	109.5 (3)	C12—C13—C14—C15	−0.2 (4)
C22—N1—C5—C6	−109.2 (2)	C21—O4—C15—C14	−164.6 (3)
C1—N1—C5—C6	82.0 (3)	C21—O4—C15—C16	16.4 (4)
C22—N1—C5—C4	119.1 (2)	C13—C14—C15—O4	179.3 (2)
C1—N1—C5—C4	−49.7 (3)	C13—C14—C15—C16	−1.7 (4)
C3—C4—C5—N1	55.9 (3)	O4—C15—C16—C17	−179.0 (3)
C19—C4—C5—N1	−64.8 (3)	C14—C15—C16—C17	2.1 (4)
C3—C4—C5—C6	−72.5 (3)	C15—C16—C17—C12	−0.6 (5)
C19—C4—C5—C6	166.8 (2)	C13—C12—C17—C16	−1.3 (4)
N1—C5—C6—C11	58.9 (3)	C1—C12—C17—C16	−178.3 (3)
C4—C5—C6—C11	−174.3 (2)	C5—N1—C22—O1	7.8 (4)
N1—C5—C6—C7	−124.5 (3)	C1—N1—C22—O1	176.2 (2)
C4—C5—C6—C7	2.3 (4)	C5—N1—C22—C23	−172.4 (2)
C11—C6—C7—C8	−1.8 (4)	C1—N1—C22—C23	−4.0 (4)
C5—C6—C7—C8	−178.4 (3)	O1—C22—C23—Br1	−98.9 (3)
C6—C7—C8—C9	1.1 (5)	N1—C22—C23—Br1	81.2 (3)

### Hydrogen-bond geometry (Å, °)

**Table:**

*D*—H···*A*	*D*—H	H···*A*	*D*···*A*	*D*—H···*A*
C1—H1···Br1	0.98	2.82	3.523 (3)	129
C20—H20C···O1^i^	0.96	2.60	3.357 (7)	136

Symmetry codes: (i) *x*−1, −*y*+1, *z*−3/2.
